# Determinación y determinantes sociales de la malaria: revisión sistemática, 1980-2018

**DOI:** 10.26633/RPSP.2019.39

**Published:** 2019-04-17

**Authors:** Jaiberth Antonio Cardona-Arias, Walter Alfredo Salas-Zapata, Jaime Carmona-Fonseca

**Affiliations:** 1 Universidad de Antioquia Universidad de Antioquia Escuela de Microbiología Medellín Colombia Escuela de Microbiología, Universidad de Antioquia, Medellín, Colombia.; 2 Universidad de Antioquia Universidad de Antioquia Grupo Salud y Comunidad César Uribe Piedrahíta Medellín Colombia Grupo Salud y Comunidad César Uribe Piedrahíta, Universidad de Antioquia, Medellín, Colombia.

**Keywords:** Determinantes sociales de la salud, malaria, medicina social, Social determinants of health, malaria, social medicine, Determinantes sociais da saúde, malária, medicina social

## Abstract

**Objetivo.:**

Describir cómo se ha aplicado el enfoque de la determinación social de la salud en los estudios sobre malaria en el mundo.

**Métodos.:**

Revisión sistemática de los estudios originales publicados entre 1980 y 2018. Se emplearon seis estrategias de búsqueda en diez bases de datos multidisciplinarias, y en las bibliotecas y los repositorios de siete universidades de Brasil, Colombia, Ecuador y Perú. Se siguió la guía PRISMA; la calidad metodológica se evaluó según los criterios de STROBE y se realizó la síntesis cualitativa de los resultados.

**Resultados.:**

Diez estudios publicados entre 1984 y 2017 cumplieron con los criterios de inclusión y exclusión preestablecidos; se identificaron 33 determinantes sociales de la malaria. De los determinantes individuales se halló mayor riesgo de malaria en adultos, personas con hábitos nocturnos y sin prácticas preventivas; de los intermedios, fueron las viviendas con mala infraestructura física y sanitaria, hacinamiento, ubicadas en áreas boscosas y con animales. De los socioeconómicos, el mayor riesgo correspondió a personas con actividades agroforestales, migrantes, y con bajos ingresos y escolaridad. La malaria ocasionó elevadas pérdidas económicas y generó pobreza y retardo educativo.

**Conclusión.:**

No se hallaron estudios con los enfoques de la Medicina Social Latinoamericana ni que aplicaran el análisis jerárquico y multinivel para los determinantes individuales, intermedios y estructurales, de la Organización Mundial de la Salud. No se ha logrado avanzar en el análisis de categorías sociales —territorio, clase social, género, etnia, políticas macroeconómicas— u otras características socioeconómicas que determinan el riesgo de enfermar o morir de malaria.

## Abstract (en)

**Objective.:**

Describe how the ‘social determination of health’ approach has been applied in malaria studies around the world.

**Methods.:**

Systematic review of original studies published from 1980 to 2018. Six search strategies were used in ten multidisciplinary databases, and in libraries and repositories of seven universities in Brazil, Colombia, Ecuador, and Peru. PRISMA guidelines were followed, methodological quality was evaluated according to STROBE criteria, and a qualitative summary of the results was conducted.

**Results.:**

Ten studies published from 1984 to 2017 met pre-established inclusion and exclusion criteria; 33 social determinants of malaria were identified. Of individual determinants, greater malaria risk was found in adults, people who are often outdoors at night, and people who do not take preventive measures; intermediate determinants were dwellings with poor physical and sanitary infrastructure, overcrowded, located in forested areas, and containing animals. Regarding socioeconomic determinants, the people at greatest risk were involved in agro-forestry activities, migrants, and those with low income and a low educational level. Malaria caused high economic losses and led to poverty and educational delay.

**Conclusion.:**

No studies were found that used Latin American social medicine approaches or that applied the World Health Organization’s hierarchical and multilevel analysis for individual, intermediate, and structural determinants. No progress has been made in the analysis of social categories—territory, social class, gender, ethnic group, macroeconomic policies—or other socioeconomic characteristics that determine risk of illness or death from malaria.

## RESUMO

**Objetivo.:**

Descrever como foi aplicado o enfoque da determinação social da saúde nos estudos de malária no mundo.

**Métodos.:**

Uma revisão sistemática de estudos originais publicados entre 1980 e 2018 foi conduzida com o uso de seis estratégias de busca em 10 bases de dados multidisciplinares e em bibliotecas e repositórios de sete universidades do Brasil, Colômbia, Equador e Peru. O estudo se baseou nos critérios da recomendação PRISMA e a qualidade metodológica foi avaliada segundo os critérios da iniciativa STROBE. Foi realizada uma síntese qualitativa dos resultados.

**Resultados.:**

Dez estudos publicados entre 1984 e 2017 preencheram os critérios de inclusão e exclusão predefinidos. Foram identificados 33 determinantes sociais da malária. Com relação aos determinantes individuais, foi observado maior risco da malária em adultos, pessoas com hábitos noturnos e que não aderem a práticas preventivas. Os determinantes intermediários foram moradias com infraestruturas física e sanitária precárias, com aglomeração de pessoas, localizadas em áreas de mata e com a presença de animais. E, quanto aos determinantes socioeconômicos, houve maior risco da doença entre os indivíduos que se dedicam a atividades agroflorestais, migrantes e pessoas com baixa escolaridade e baixa renda. A malária causou grande prejuízo econômico, gerando pobreza e atraso educacional.

**Conclusão.:**

Não foram encontrados estudos com enfoques da medicina social latino-americana nem estudos contendo análises com modelo hierárquico e multinível para os determinantes individuais, intermediários e estruturais de acordo com a definição da Organização Mundial da Saúde. Não foi possível aprofundar a análise das categorias sociais (território, classe social, gênero, etnia e políticas macroeconômicas) ou outras características socioeconômicas que determinam o risco de ter a doença ou morrer de malária.

La malaria es una de las principales enfermedades infecciosas en el mundo; pese a ser prevenible y curable, presenta elevadas tasas de morbilidad y mortalidad, con 216 millones de casos y 445 000 muertes (70% de ellas en menores de cinco años) en el año 2016 (1). Desde el año 2000, las Naciones Unidas hizo explícito el compromiso de combatir esta enfermedad en los Objetivos de Desarrollo de Milenio (1, 2), y su control debe contribuir al logro de los objetivos de la Agenda 2030 sobre Desarrollo Sostenible (3). En el marco de la Estrategia Técnica Mundial contra la Malaria 2016-2030, en el 2016 se invirtieron US$ 2 700 millones, un esfuerzo de la Organización Mundial de la Salud (OMS) para reducir en 90% la incidencia y la mortalidad por esta enfermedad, eliminarla en 35 países y garantizar el acceso universal a servicios de prevención, diagnóstico y tratamiento (1, 4).

En este contexto, los programas de control palúdico parecen haber dejado a un lado el hecho de que el riesgo de enfermar y morir por esta enfermedad está relacionado con las condiciones socioeconómicas (5-7) —también descritas como “variables sociales”— como la escolaridad de la madre (8), los ingresos familiares (9), las condiciones de vida “subóptimas” que implican riesgo vectorial (10, 11), el hacinamiento (12), el inadecuado acceso a cuidados de salud (13, 14) y el tipo de sistema de salud, entre otras variables sociales, ambientales y económicas (15-17). Está aceptado que esta infección enlentece el avance social, al afectar más a poblaciones con problemas socioeconómicos (18, 19). De hecho, se ha afirmado que la malaria constituye una “trampa de la pobreza”, en la medida que esta aumenta la vulnerabilidad y agudiza la pobreza en las familias (20).

A pesar de lo anterior, en las investigaciones sobre la malaria predominan los estudios biomédicos y no es frecuente hallar estudios sobre su relación con las condiciones o los estilos de vida. En general, existe poca experiencia en el estudio de los determinantes sociales de la salud (DSS) —planteados por la OMS y asumidos por la Organización Panamericana de la Salud (OPS)— y de la determinación social —de la Medicina Social Latinoamericana (MSL)—, aplicados a esta enfermedad.

Según la OMS, los DSS “son las circunstancias en que las personas nacen, crecen, viven, trabajan y envejecen”, acorde con “la distribución del dinero, el poder y los recursos a nivel mundial, nacional y local*”,* y explican las desigualdades relacionadas con la salud. Los DSS se agrupan en individuales (conductuales y biológicos), intermedios (trabajo, ingresos, situación económica, vivienda y entorno) y estructurales (políticas macroeconómicas, clase, género, etnia y territorio) (21). Por su parte, la MSL define los procesos de determinación social de la salud como las relaciones de poder, las dinámicas de acumulación de capital y los patrones de consumo, trabajo y desgaste social, que definen las diferencias en los procesos salud-enfermedad según la clase social, el género y la etnia (22, 23).

Estos dos enfoques de determinantes sociales coinciden en destacar la relevancia de las condiciones socioeconómicas, las políticas públicas, la pobreza y las condiciones laborales, para explicar los perfiles epidemiológicos y la interacción biología-sociedad. Sin embargo, ambos enfoques presentan discrepancias en varios aspectos: las formas de explicar la causalidad y el riesgo; la concepción de lo social como suma de individuos o como realidad irreductible-dinámica; la perspectiva funcionalista o dialéctica de lo social; el concepto de clase social como *estatus* educativo, de ingresos u ocupación, o como gradientes derivados de relaciones de poder y la acumulación del capital; y la focalización en la medición de desigualdades relacionadas con la salud o en los procesos de generación de inequidades sanitarias (24).

No obstante los avances experimentados en los diversos enfoques, aún se requiere mayor investigación sobre los DSS relacionados con la malaria. Por ello, se diseñó esta investigación con el objetivo de describir cómo se ha aplicado el enfoque de la determinación social de la salud en los estudios sobre malaria en el mundo.

## MATERIALES Y MÉTODOS

Se realizó una revisión sistemática de la literatura, ajustada a las recomendaciones contenidas en la guía PRISMA (Preferred Reporting Items for Systematic Reviews and Meta-Analyses) (25).

### Estrategia de selección de los artículos

Se realizó una búsqueda sistemática de los artículos publicados entre 1980 y 2018, en cuatro etapas:

#### Identificación.

Se efectuaron búsquedas en 10 bases de datos: Pubmed-Medline, SciELO, Science-Direct, Scopus, Jstor, Web of Science, EBSCO-Host, HAPI, Google Scholar y Malaria in Pregnancy Library. La búsqueda se extendió al sistema de bibliotecas y los repositorios institucionales de siete universidades: de Antioquia, de los Andes, del Valle y Nacional (todas de Colombia); Andina Simón Bolívar (de Ecuador), Federal de Amazonas (de Brasil) y Nacional Mayor de San Marcos (Perú).

En todos los casos (bases de datos, bibliotecas y repositorios) se aplicaron seis estrategias de búsqueda mediante combinaciones de palabras clave: i) social determination & malaria, ii) social determination & Plasmodium, iii) social determination & paludism, iv) social determinants & malaria, v) social determinants & Plasmodium y vi) social determinants & paludism; para un total de 102 búsquedas. Estas estrategias y descriptores —identificados mediante la técnica denominada “bola de nieve” o “*pearl growing*” (26) y según los tesauros DeCS y MeSH— se ajustaron según las especificidades de las fuentes y el idioma. Los detalles de las estrategias específicas pueden solicitarse a los autores.

#### Tamizaje.

A los artículos identificados se aplicaron dos criterios de inclusión: el primero, ser estudios originales, sin restricciones por el tipo de investigación o el diseño empleado; así se descartaron los artículos de revisión, los editoriales y los libros. Como segundo criterio de inclusión, los artículos debían tener como objetivo central estudiar la malaria.

#### Selección.

Se excluyeron los estudios que no mencionaran explícitamente los determinantes sociales de la malaria, en los que la malaria era un determinante de otros eventos —como la salud materno-infantil o la deserción escolar— y los estudios no disponibles en texto completo. Se revisaron las referencias bibliográficas de los estudios seleccionados a fin de hacer la búsqueda más exhaustiva.

#### Inclusión.

Con los artículos incluidos, se realizó una síntesis cualitativa de las variables título, autores, años de publicación y de realización del estudio, país, tipo de estudio, y población y determinantes estudiados.

### Reproducibilidad y calidad metodológica

Para garantizar la reproducibilidad en la búsqueda, la selección y la extracción de la información, dos investigadores aplicaron las cuatro fases de la búsqueda sistemática y extrajeron los datos a un documento de MS Excel diseñado para el caso; las discrepancias se remitieron al tercer autor y se resolvieron por consenso. La calidad metodológica se evaluó según las pautas contenidas en la guía STROBE (Strengthening the Reporting of Observational Studies in Epidemiology), que incluyen criterios de validez interna y externa (27).

### Análisis de la información

Se determinó la frecuencia de las variables estudiadas y de los determinantes (demográficos, de salud, del hogar, sociales, económicos, y de la comunidad y el ambiente) incluidos en los artículos analizados; se calculó el porcentaje de estudios que cumplían con cada uno de los criterios de la guía STROBE.

## RESULTADOS

**FIGURA 1:**
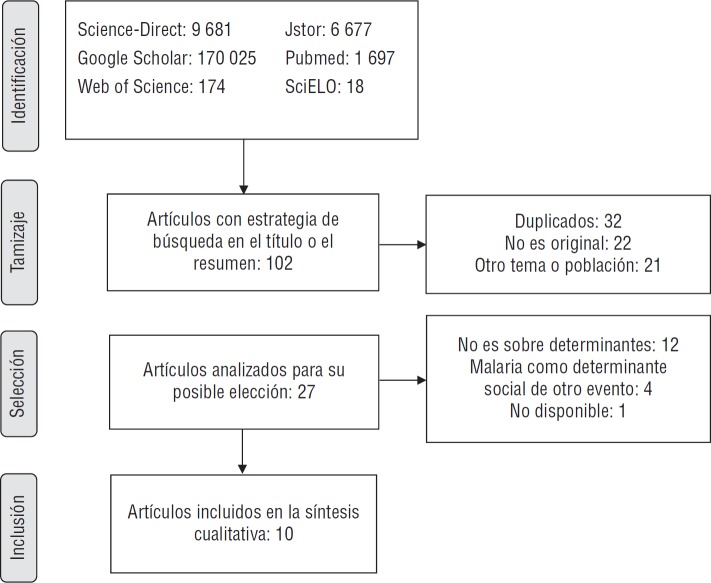
Flujograma de búsqueda y selección de los estudios sobre determinantes sociales de la malaria

Se identificaron 188 272 textos; de ellos, 102 contenían los términos de búsqueda en el título o el resumen, pero solo 10 aludían de manera explícita a los DSS relacionados con la malaria (figura 1). En las bases de datos EBSCO Host, HAPI, Malaria in Pregnancy Library, las bibliotecas, los repositorios y las referencias de los artículos seleccionados no se hallaron estudios que cumplieran los criterios de selección.

Ningún estudio se desarrolló bajo el enfoque de la determinación social de la salud de la MSL y, a pesar de usar el término “determinantes sociales”, en ninguno se aplicó estrictamente el modelo de los DSS de la OMS/OPS. Las publicaciones aplicaron diseños propios de la epidemiología clásica: cinco eran estudios transversales (28-32), dos eran analíticos o de comparación de grupos (19, 33) y tres eran ecológicos con unidades geográficas o temporales (18, 34, 35).

De los 10 estudios seleccionados, solo dos salieron a la luz en las décadas de 1980 y 1990, mientras los restantes se publicaron a partir del año 2000, fecha en que se presentaron cambios en la epidemiología clásica y se consolidaron los dos enfoques de la determinación social de la salud. Con relación a los países en los que se realizaron las investigaciones, cuatro se desarrollaron en África, tres en América y tres en Asia.

Los estudios utilizaron diferentes unidades de análisis: cuatro investigaciones se realizaron en hogares (*n* = 1 626 casos en total); una se basó en los registros de *Plasmodium falciparum* (*n* = 18 034) y *P. vivax* (*n* = 5 178); una comparó personas con malaria (*n* = 58) y sin malaria (*n* = 58), y las cuatro restantes estudiaron determinantes sociales en 2 236 personas de zonas endémicas de malaria, según su posición en la familia, como jefes del hogar (*n* = 471), madres (*n* = 738), menores de cinco años (*n* = 647) y que trabajaban como mineros (*n* = 380) (cuadro 1).

**FIGURA 2:**
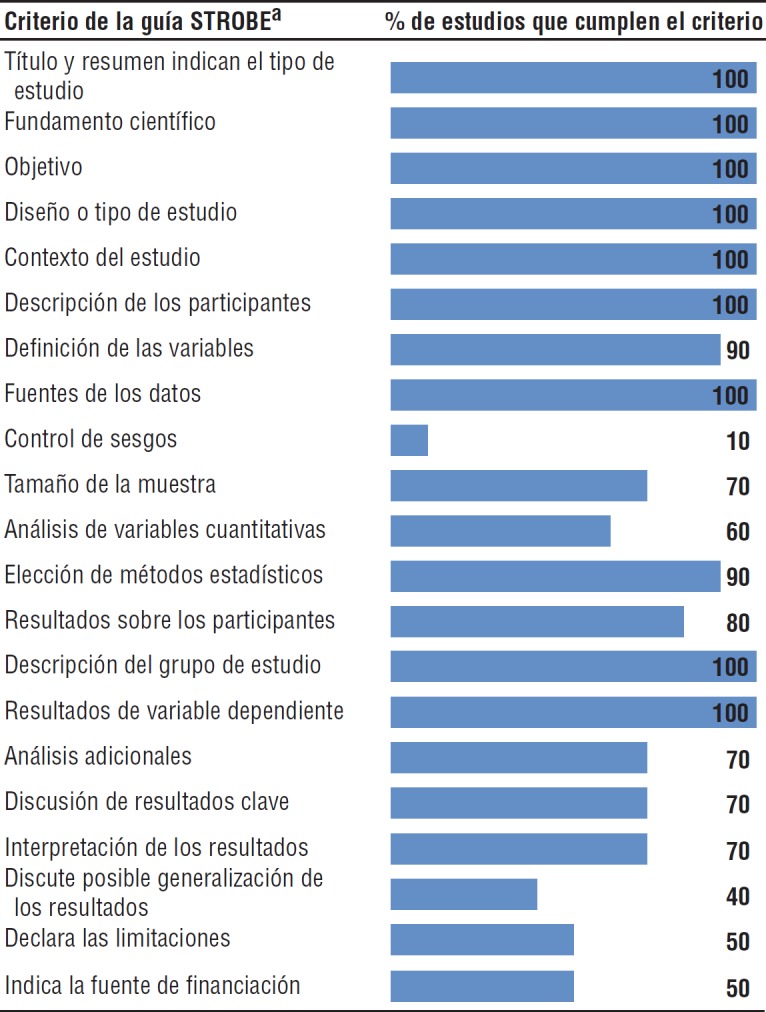
Evaluación de la calidad metodológica

La calidad metodológica de los estudios fue buena, ya que todos cumplieron con más de 65% de los criterios de la guía STROBE, con excepción del estudio de Banguero (33) que cumplió con 57%. Los criterios menos aplicados fueron el control de sesgos (10%), la generalización de resultados (40%), la declaración de las limitaciones (50%) y la develación de las fuentes de financiación (50%) (figura 2).

En total, se identificaron 33 DSS. De los individuales, los más frecuentes fueron: el diagnóstico previo de malaria, en tres estudios (28, 31, 33), y la medición de conocimientos actitudes y prácticas sobre la enfermedad, en cuatro (29, 31, 33, 34). De los determinantes del hogar, el más frecuente fue el de los factores de riesgo del domicilio y el peridomicilio, en cuatro estudios (19, 29, 32, 33). Entre los determinantes socioeconómicos, los más frecuentemente estudiados fueron: el nivel educativo, en cinco estudios (18, 30-33) (principalmente de la madre), y los ingresos financieros, en siete (18, 19, 28, 30, 32, 33, 35) (cuadro 2). Solamente 21 de los DSS estudiados presentaron asociaciones estadísticas, pero no fue posible realizar una síntesis cuantitativa debido a la heterogeneidad de los cálculos y los sujetos evaluados (niños, madres, jefes de hogar, etc.), y las medidas estadísticas empleadas (pruebas de hipótesis, razones de posibilidades —u *odds ratios—* y coeficientes de regresión, entre otros).

En los determinantes individuales, se halló mayor riesgo de malaria en los adultos (28-30, 33), las personas con hábitos nocturnos (19) y las que no aplicaban medidas preventivas, tales como el uso de mosquiteros e insecticidas (19, 29). De los determinantes intermedios, la mayor frecuencia de la enfermedad se registró en las viviendas con mala infraestructura física (materiales de construcción precarios) y sanitaria (fuente inadecuada de agua de consumo e incorrecta eliminación de desechos), con hacinamiento, con niños menores de 5 años (19, 29, 31-33), ubicadas en áreas boscosas (35) y con presencia de animales (29). En cuanto a los determinantes socioeconómicos, el mayor riesgo se registró en las personas dedicadas a actividades agroforestales (31, 33) o que trabajaban en instalaciones con poco uso del insecticida DDT, mosquiteros y medicamentos profilácticos o terapéuticos (19, 33).

***Fuente:*** Elaboración propia a partir de datos publicados.

**CUADRO 1:** Descripción de los estudios analizados sobre determinantes sociales de la malaria

Autor	Año de publicación (período analizado)	País	Muestra
Banguero (33)	1984 (1980-1982)	Colombia	217 hogares con malaria y 217 sin malaria
Vosti (28)	1990 (1984)	Brasil	380 mineros de cuatro minas^a^
Yadav y cols. (34)	2005 (1999-2003)	India	300 hogares^a^
Thuilliez (18)	2010 (2001-2006)	Malí	403 hogares en 2001 y 407 hogares en 2006^a^
Bui y cols. (35)	2011 (2007-2008)	Vietnam	18 034 casos de *P. falciparum* y 5 178 de *P. vivax*
Sánchez y Chamizo (29)	2012 (2007)	Costa Rica	82 hogares^a^
Chukwuocha y cols. (30)	2013 (2013)	Nigeria	738 madres de cuatro comunidades rurales^a^
Aristianti y cols. (19)	2014 (2012)	Indonesia	58 personas con malaria y 58 controles
Shayo y cols. (31)	2015 (2012)	Tanzania	471 jefes de hogar de cuatro aldeas^a^
Ma y cols. (32)	2017 (2010)	República Democrática del Congo	647 menores de 5 años

***Fuente:*** Elaboración propia a partir de datos publicados.

a Se estudió la población expuesta, con y sin malaria.

También se constataron elevadas pérdidas económicas por días de inactividad u hospitalización por malaria: aproximadamente 10 días por cada episodio de la enfermedad (28, 35). Algunos autores consideraron la malaria como un *proxy* de la pobreza y como un factor asociado con el retardo en el logro educativo, y encontraron una relación de influencia mutua entre la enfermedad y el progreso social: una mayor prevalencia de malaria aumenta la probabilidad de repetición escolar y disminuye los ingresos personales y familiares, al tiempo que los bajos niveles de ingreso y de escolaridad derivan en un mayor número de casos de malaria e impiden o retrasan la búsqueda de tratamiento (18, 30-32, 35). Esto implica que la atención y el control de la enfermedad pueden contribuir a mejorar los indicadores educativos y socioeconómicos, y la intervención de estos últimos pueden contribuir a la reducción de las tasas de morbilidad y mortalidad por malaria (30-32).

La síntesis cualitativa de los artículos analizados reveló que no se ha logrado avanzar en el análisis de categorías sociales —como el territorio, la clase social, el género, la etnia, las políticas macroeconómicas y otras variables sociales— que inciden en las tasas de morbilidad y mortalidad de la malaria. Esto puede indicar que los cambios de paradigmas para la investigación social de la malaria requieren de tiempos prolongados y de un mayor compromiso político de las agencias internacionales y los gobiernos locales.

***Fuente:*** Elaboración propia a partir de datos publicados.

^a^ Strengthening the Reporting of Observational Studies in Epidemiology (27).

**CUADRO 2:** Determinantes sociales de la salud relacionados con la malaria estudiados en los artículos analizados

Estudio	Determinantes individuales			Determinantes intermedios		
	Demográficos	Estado de salud	Domicilio	Sociales	Económicos	Comunidad y ambiente
Banguero (33)	Sexo, edad, relación con el cabeza del hogar, estado civil, fertilidad	Nutrición, estado de salud, antecedentes de malaria, infección actual, vacunación	Composición, relaciones familiares, conocimientos y actitudes hacia la malaria	Educación, actividades no económicas	Fuente de ingreso, riqueza y ocupación, empleo, condiciones materiales de la vivienda	Disponibilidad de servicios públicos^a^ y de personal de salud; organización comunitaria y de salud
Vosti (28)	Edad, residencia (urbana *vs.* rural) y tipo de mina	Exposición a malaria y episodios previos	Casos previos de malaria en la familia	SD^b^	Nivel de ingresos	SD
Yadav y cols. (34)	SD	Conocimientos sobre causas y efectos clínicos de la malaria	Conocimientos y actitudes hacia la prevención y el tratamiento	Migración	SD	SD
Thuilliez (18)	SD	Salud del niño^c^	SD	Logro escolar	Índice de estatus socioeconómico, ingreso, nivel de riqueza	SD
Bui y cols. (35)	SD	SD	Participación en actividades de control	Migración	Población pobre	Porcentaje del distrito cubierto por bosques; precipitaciones, temperatura, acceso a servicios de salud
Sánchez y Chamizo (29)	Características demográficas	Percepciones y prácticas preventivas	Ambiente doméstico y peridoméstico, convivencia con animales	SD	Ocupación y vivienda (tenencia y condiciones materiales)	SD
Chukwuocha y cols. (30)	Edad, paridad, estado civil	SD	SD	Nivel educativo	Ocupación y estatus socioeconómico según ingresos	SD
Aristianti y cols. (19)	SD	Hábitos nocturnos y comportamientos preventivos	Condiciones del hogar, ambiente y trabajo	Migración	Condiciones materiales de la vivienda y condición socioeconómica	SD
Shayo y cols. (31)	Edad y sexo	Antecedentes de malaria; conocimientos, prácticas y prevención	SD	Nivel educativo	Fuente se sustento	Distancia al centro de salud, acceso a tratamiento
Ma y cols. (32)	Datos demográficos	Uso de mosquitero y enfermedad febril en el último mes	Composición del hogar: número de personas y niños menores de 5 años	Educación materna	Nivel de riqueza, bienes del hogar^d^ y tipo de vivienda^e^; mosquiteros	Calidad de la atención médica accedida

***Fuente:*** Elaboración propia a partir de datos publicados.

a Agua, electricidad, acueducto, correo, centro de salud y escuelas.

b SD: sin datos disponibles.

c Vacunación, suplemento de vitamina A, diarrea, fiebre, variables antropométricas y tratamientos.

d Vehículos automotores, electricidad, radio, televisión, teléfono, refrigerador.

e Ladrillo y mortero *vs.* paja.

## DISCUSIÓN

En esta revisión se analizaron artículos científicos publicados en las últimas cuatro décadas, que abarcaron un elevado número de hogares y personas expuestas a la malaria, con una gran heterogeneidad en las poblaciones evaluadas y los DSS estudiados. La metodología seguida (36) permitió evaluar y sintetizar los resultados de varios estudios, identificar tendencias, consolidar hipótesis y generar resultados útiles para muy diversos grupos; además, se identificaron DSS que podrían emplearse en nuevas investigaciones dirigidas a estudiar el complejo mundo de la malaria.

De los estudios analizados, 70% se realizaron en África y América, donde la situación es más grave. A los países africanos correspondió el 90% de la morbilidad y la mortalidad en 2016 (1), mientras que en América la mortalidad estimada entre 1991 y 2009 fue de 6 personas por 100 000 habitantes, según un metaanálisis con datos de 21 países (37).

Los estudios, publicados entre 1984 y 2017, se hicieron más frecuentes a partir de finales del siglo XX, cuando se adoptaron los enfoques de los DSS de la OMS/OPS y de la determinación social de la MSL; el primero, articulado con la epidemiología social anglosajona y el segundo, con la salud colectiva y la epidemiología crítica. Este avance convergió con cambios en los modelos de desarrollo económico, la configuración de los Estados de bienestar y la adopción de la atención primaria como modelo de salud, durante las décadas de 1970 y 1980 (24). Pese al amplio período analizado, el número de estudios sobre DSS relacionados con la malaria resultó bajo, algo en lo que coincide otra revisión sistemática enfocada en los determinantes sociales del parasitismo intestinal, la anemia y la malnutrición (38).

Por otra parte, los estudios analizados utilizaron diseños propios de la epidemiología clásica, que no guardan relación con la perspectiva multinivel que plantea el enfoque de DSS de la OMS/OPS, lo que demuestra un rezago importante en la aplicación de estas ideas en los estudios de malaria (39). En ese sentido, vale precisar que los DSS se pueden medir en unidades agregadas o individuales, pero la inferencia que se haga a los individuos o los grupos requiere de diseños multinivel o la unificación de individuos pertenecientes a grupos diferentes, a fin de evitar las falacias ecológica y atomista y evidenciar cómo las variables socioeconómicas afectan al dominio individual (40).

Pese a la ausencia de análisis multinivel, se constató que las migraciones, las condiciones materiales de la vivienda, y el nivel educativo y de ingreso fueron los principales DSS relacionados con la malaria. La migración ha permitido explicar los casos importados por el turismo en Europa y los asociados con poblaciones de refugiados —por el narcotráfico, el terrorismo o la violencia política— en África y América Latina. El efecto de las migraciones se hace más grave por los problemas concomitantes de bajos ingresos económicos, dificultades para conseguir alimentos, insuficiente conocimiento de la enfermedad y problemas de acceso a acciones de prevención y al tratamiento (41-44).

Las condiciones materiales de la vivienda constituyen un factor de riesgo importante de malaria: estudios de casos y controles han mostrado el riesgo asociado a paredes de estera y de adobe sin enlucir y a áreas sin techo, así como a la cercanía de ríos, acequias y cultivos de frutales (45). Además, la vivienda es un DSS intermedio en el modelo de la OMS/OPS (21, 39) y un indicador de la situación económica de la familia; constituye, además, un medio para satisfacer necesidades biológicas, psicológicas y sociales, y es uno de los primeros escenarios sobre los que se puede actuar para mitigar el riesgo de enfermar y para promocionar la salud (46).

En relación con la escolaridad y los ingresos, algunos estudios informan pérdidas económicas por la malaria y consideran esta enfermedad como un *proxy* de la pobreza y el retardo escolar. En tal sentido, los DSS de la OMS/OPS plantean que las personas “llegan a diferentes posiciones en esta estratificación dependiendo de su clase social, su estatus ocupacional, su nivel educativo y sus ingresos”. El estatus y la clase social determinan el comportamiento y las condiciones de vida e influyen en el desenlace de los problemas de salud (47, 48).

Al hacer este análisis, se debe tener en cuenta que el estatus corresponde a jerarquías basadas en el prestigio social de algunas ocupaciones, una estratificación social en función del ingreso o la escolaridad, sin develar los mecanismos sociales que derivan en un acceso diferencial de las personas a los recursos económicos, educativos, políticos o culturales. Por su parte, la clase social tiene mayor alcance explicativo, al dar cuenta de las relaciones de propiedad de los recursos productivos que derivan en diferentes niveles de ingreso, educación y calidad de vida en general (49).

En el caso de investigaciones sobre los DSS relacionados con la malaria, predomina la perspectiva del estatus, lo que ha redundado en una baja comprensión de las causas sociales de las desigualdades y las inequidades que afectan a la salud (47, 50). Esto se ha debido a que los determinantes sociales sistematizados no dan cuenta de constructos sociales sino de reducciones en algunas variables.

No se han encontrado estudios que trasciendan las relaciones estadísticas hacia la causalidad sociológica, histórica o cultural. La investigación sociocultural e histórica de la malaria es imprescindible para comprender las concepciones sobre la enfermedad, los saberes y prácticas de prevención y control, la necesidad de buscar tratamientos, entre otros aspectos que redundan en acciones individuales y colectivas que condicionan los resultados de los programas de control (51).

Además de las diferencias derivadas del uso del estatus social o de la clase social, surgen otras discrepancias derivadas de los enfoques propugnados por la OMS/OPS y la MSL sobre los determinantes de la malaria. El enfoque de la MSL entraña importantes diferencias que se deben tomar en cuenta al investigar los DSS relacionados con la malaria, en particular: i) considera la epidemiología como una disciplina que no evade el compromiso con la transformación social e incorpora el análisis de las relaciones de poder; ii) no concibe los DSS estructurales o intermedios como algo distante al individuo —o como una realidad externa de la cual las personas reciben una exposición diferencial a factores de riesgo—, sino como una realidad concreta; iii) comprende los modos y condiciones de vida como procesos históricos y no como una relación probabilística con el estado de salud; y iv) hace explícitas las diferencias históricas de las nociones de justicia y equidad sanitarias (24).

El escaso uso del enfoque de la OMS/OPS sobre los DSS en los artículos analizados debe llevar a la reflexión sobre los tiempos requeridos para la introducción de cambios en los paradigmas epidemiológicos clásicos para la investigación social de la malaria. Por su parte, la ausencia de estudios basados en el enfoque de la MSL se puede atribuir a la hegemonía de la epidemiología positivista, de mayor facilidad analítica, así como a las dificultades que impone el trabajo interdisciplinario, la necesidad de innovaciones teórico-conceptuales y metodológicas para relacionar las categorías sociales con los desenlaces de la salud y la baja capacidad de atraer financiamiento; otra explicación podría ser el escaso interés de los investigadores por estos enfoques (52).

Entre las principales limitaciones del presente estudio, se debe mencionar el sesgo que podrían introducir algunas investigaciones sobre la malaria —principalmente de las décadas de 1980 y 1990—, que no tomaron en cuenta la relación de algunas condiciones sociales y económicas que inciden en la enfermedad. Sin embargo, en el protocolo de búsqueda y selección empleado no se aplicaron restricciones por el tipo o el diseño del estudio, por lo que esta variable no debe haber influido en los resultados y las conclusiones. El incipiente desarrollo metodológico de los enfoques de los DSS relacionados con la malaria impide visibilizar las relaciones entre diferentes niveles de determinación y las categorías más relevantes en cada uno de ellos.

En conclusión, se comprobó que en las bases de datos bibliográficas no hay investigaciones sobre malaria que apliquen el enfoque de la determinación social según la MSL; tampoco se encontraron artículos que aplicaran el análisis jerárquico y multinivel de los DSS individuales, intermedios o estructurales de la OMS/OPS. Se observó un predominio del enfoque empírico e individual, sin desarrollo conceptual y metodológico para las categorías comunitarias, sociales o estructurales de la determinación social de la salud. Esto pone de manifiesto la necesidad de emprender estudios multinivel y la importancia de propiciar cambios paradigmáticos en las investigaciones sobre salud pública tradicional, mediante acuerdos entre los investigadores en este campo, la acción de las agencias internacionales que financian investigaciones relacionadas con la salud pública y los gobiernos, con el fin de lograr un impacto global e integral en los países donde la malaria sigue siendo endémica.

Los estudios sobre malaria deben tomar en cuenta las categorías sociales —políticas macroeconómicas, mercado laboral, relaciones de poder, causas de la pobreza, clase social y territorio— y analizar la temporalidad de los fenómenos macrosociales, cuyos cambios se evidencian en períodos más prolongados que los fenómenos del orden microsocial. Se deben explicar las relaciones existentes entre el gradiente socioeconómico y la malaria, sin caer en la falacia atomista y ecológica, e incluir el análisis histórico, ético, político y económico de los procesos de determinación social de las inequidades sanitarias relacionadas con la malaria.

## Contribución de los autores.

JACA concibió el estudio, planificó, recolectó y analizó los datos y escribió el manuscrito. Todos los autores interpretaron los resultados, revisaron el manuscrito y aprobaron la versión final.

## Apoyos financieros:

El trabajo recibió financiamiento de Colciencias (proyecto 111577757051, contrato 755-2017) y de la Estrategia de Sostenibilidad CODI, Universidad de Antioquia 2016-2017 (código ES-84160127). Los autores declaramos que estas instancias no participaron de ninguna manera en el diseño del estudio, la colecta y análisis de los datos, la decisión de publicar este trabajo ni la preparación del manuscrito.

## Declaración.

Las opiniones expresadas en este manuscrito son únicamente responsabilidad de los autores y no reflejan necesariamente los de la *Revista Panamericana de Salud Pública* o la Organización Panamericana de la Salud.

## References

[1] 1. Organización Mundial de la Salud. Nota descriptiva: paludismo. Ginebra: OMS; 2018 [citado el 4 de marzo de 2019]. Disponible en: http://www.who.int/es/news-room/fact-sheets/detail/malaria

[2] 2. Naciones Unidas, Asamblea General. Declaración del Milenio. (Resolución A/RES/55/2). Nueva York: NU; 2000 [citado el 4 de marzo de 2019]. Disponible en: http://www.un.org/spanish/milenio/ares552.pdf.

[3] 3. Naciones Unidas. Objetivos de Desarrollo Sostenible, 17 objetivos para transformar nuestro mundo. Nueva York: NU; 2018 [citado el 4 de marzo de 2019]. Disponible en: https://www.un.org/sustainabledevelopment/es

[4] 4. Organización Mundial de la Salud. Programa Mundial sobre Paludismo de la OMS. Ginebra: OMS; 2017 [citado el 4 de marzo de 2019]. Disponible en: http://www.who.int/malaria/about_us/es/

[5] 5. Roberts B, Odong VN, Browne J, Ocaka KF, Geissler W, Sondorp E. An exploration of social determinants of health amongst internally displaced persons in northern Uganda. Confl Health. 2009;3:10. 10.1186/1752-1505-3-10 PMC2803159 20003516

[6] 6. Braveman P. Accumulating knowledge on the social determinants of health and infectious disease. Public Health Rep. 2011;126(Suppl 3):28–30. 10.1177/00333549111260S306 PMC3150126 21836734

[7] 7. Marmot M, Friel S, Bell R, Houweling TA, Taylor S. Commission on Social Determinants of Health. Closing the gap in a generation: health equity through action on the social determinants of health. Lancet. 2008;372(9650):1661–9. 10.1016/S0140-6736(08)61690-6 18994664

[8] 8. Njau JD, Stephenson R, Menon MP, Kachur SP, McFarland DA. Investigating the important correlates of maternal education and childhood malaria infections. Am J Trop Med Hyg. 2014;91(3):509–19. 10.4269/ajtmh.13-0713 PMC4155551 25002302

[9] 9. Graves PM, Richards FO, Ngondi J, Emerson PM, Shargie EB, Endeshaw T, et al. Individual, household and environmental risk factors for malaria infection in Amhara, Oromia and SNNP regions of Ethiopia. Trans R Soc Trop Med Hyg. 2009;103(12):1211–20. 10.1016/j.trstmh.2008.11.016 19144366

[10] 10. Bates I, Fenton C, Gruber J, Lalloo D, Medina Lara A, Squire SB, et al. Vulnerability to malaria, tuberculosis, and HIV/AIDS infection and disease. Part 1: determinants operating at individual and household level. Lancet Infect Dis. 2004;4(5):267–77. 10.1016/S1473-3099(04)01002-3 15120343

[11] 11. Bizimana JP, Twarabamenye E, Kienberger S. Assessing the social vulnerability to malaria in Rwanda. Malar J. 2015;14:2. 10.1186/1475-2875-14-2 PMC4326441 25566988

[12] 12. Hulden L, McKitrick R, Hulden L. Average household size and the eradication of malaria. J R Stat Soc Series A. 2014;177(3):725–42.

[13] 13. Mboera LE, Kamugisha ML, Rumisha SF, Kisinza WN, Senkoro KP, Kitua AY. Malaria and mosquito net utilisation among schoolchildren in villages with or without healthcare facilities at different altitudes in Iringa District, Tanzania. Afr Health Sci. 2008;8(2):114–9. PMC2584322 19357761

[14] 14. Mboera LE, Kamugisha ML, Rumisha SF, Msangeni HA, Barongo V, Molteni E, et al. The relationship between malaria parasitaemia and availability of healthcare facility in Mpwapwa District, central Tanzania. Tanzan Health Res Bull. 2006;8(1): 22–7. 10.4314/thrb.v8i1.14266 17058796

[15] 15. Mboera L, Mazigo H, Rumisha S, Krammer R. Towards malaria elimination and its implication for vector control, disease management and livelihoods in Tanzania. Malar World J. 2013;4(19):1–14. 10.5281/zenodo.10928325 PMC11138750 38828111

[16] 16. Dillip A, Hetzel M, Gosoniu D, Kessy F, Lengeler C, Mayumana I, et al. Socio-cultural factors explaining timely and appropriate use of health facilities for degedege in south-eastern Tanzania. Malar J. 2009;8:144. 10.1186/1475-2875-8-144 PMC2712476 19563640

[17] 17. Lowassa A, Mazigo H, Mahande AM, Mwang’onde BJ, Msangi S, Mahande MJ, et al. Social economic factors and malaria transmission in Lower Moshi, northern Tanzania. Parasit Vectors. 2012;5:129. 10.1186/1756-3305-5-129 PMC3425329 22741551

[18] 18. Thuilliez J. Fever, malaria and primary repetition rates amongst school children in Mali: combining demographic and health surveys (DHS) with spatial malariological measures. Soc Sci Med. 2010;71(2):314–23. 10.1016/j.socscimed.2010.03.034 20471149

[19] 19. Aristianti V, Najmah N, Mutahar R. Social determinants of malaria in the working area of Puput Public Health Services, West Bangka. Malar World J. 2014;5(2):94–102.

[20] 20. Sachs J, Malaney P. The economic and social burden of malaria. Nature. 2002;415(6872):680–5. 10.1038/415680a 11832956

[21] 21. Organización Mundial de la Salud. Determinantes sociales de la salud. Ginebra: OMS. 2018 [citado el 4 de marzo de 2019]. Disponible en: http://www.who.int/social_determinants/es/

[22] 22. Iriart C, Waitzkin H, Breilh J, Estrada A, Merthy EE. Medicina Social Latinoamericana: aportes y desafíos. Rev Panam Salud Publica. 2002;12(2):128–36. 10.1590/s1020-49892002000800013 12243699

[23] 23. Breilh J. La epidemiología crítica: una nueva forma de mirar la salud en el espacio urbano. Salud Colectiva. 2010; 6(1):83–101.

[24] 24. Morales C, Borde E, Eslava J, Concha S. ¿Determinación social o determinantes sociales? Diferencias conceptuales e implicaciones praxiológicas. Rev Salud Publica (Bogotá). 2013;15(6):797–808. 25124346

[25] 25. PRISMA. Preferred reporting items for systematic reviews and meta-analyses (PRISMA) website [Internet]; 2015 [citado el 5 de marzo de 2019]. Disponible en: http://www.prisma-statement.org/

[26] 26. Schlosser RW, Wendt O, Bhavnani S, Nail-chiwetalu B. Use of information-seeking strategies for developing systematic reviews and engaging in evidence-based practice: the application of traditional and comprehensive pearl growing. A review. Int J Lang Community Disord. 2006;41(5):567–82. 10.1080/13682820600742190 17050471

[27] 27. Von Elm E, Altman DG, Egger M, Pocock SJ, Gøtzsche PC, Vandenbroucke JP. The Strengthening the Reporting of Observational Studies in Epidemiology (STROBE) statement: guidelines for reporting observational studies. J Clin Epidemiol. 2008;61(4):344-9. 10.1016/j.jclinepi.2007.11.008 18313558

[28] 28. Vosti S. Malaria among gold miners in southern Pará, Brazil: estimates of determinants and individual costs. Soc Sci Med. 1990;30(10):1097–105. 10.1016/0277-9536(90)90296-5 2363060

[29] 29. Sánchez Y, Chamizo H. Los determinantes socio-ambientales de la malaria en la localidad de Matina en Costa Rica. Rev Costarric Salud Publica. 2012;21(2):50–7.

[30] 30. Chukwuocha U, Okpanma A, Chukwuocha A, Dozie I. Social determinants of malaria treatment seeking time by mothers of children (0-5 years) in south eastern Nigeria. J Community Health. 2014;39(6):1171–8. 10.1007/s10900-014-9872-4 24729003

[31] 31. Shayo E, Rumisha S, Mlozi M, Bwana V, Mayala B, Malima R, et al. Social determinants of malaria and health care seeking patterns among rice farming and pastoral communities in Kilosa District in central Tanzania. Acta Trop. 2015;144:41–9. 10.1016/j.actatropica.2015.01.003 25596436

[32] 32. Ma C, Claude K, Kibendelwa Z, Brooks H, Zheng X, Hawkes M. Is maternal education a social vaccine for childhood malaria infection? A cross-sectional study from war-torn Democratic Republic of Congo. Pathog Glob Health. 2017;111(2):98–106. 10.1080/20477724.2017.1288971 PMC5375613 28220714

[33] 33. Banguero H. Socioeconomic factors associated with malaria in Colombia. Soc Sci Med. 1984;19(10):1099–104. 10.1016/0277-9536(84)90313-7 6523151

[34] 34. Yadav S, Sharma R, Joshi V. Study of social determinants of malaria in desert part of Rajasthan, India. J Vector Borne Dis. 2005;42(4):141–6. 16457383

[35] 35. Bui H, Clements A, Nguyen Q, Nguyen M, Le X, Hay S, et al. Social and environmental determinants of malaria in space and time in Viet Nam. Int J Parasitol. 2011;41(1):109–16. 10.1016/j.ijpara.2010.08.005 PMC3086784 20833173

[36] 36. Cardona A, Higuita L, Ríos L. Revisiones sistemáticas de la literatura científica. Medellín: Ediciones Universidad Cooperativa de Colombia; 2015.

[37] 37. Bardach A, Ciapponi A, Rey L, Rojas J, Mazzoni A, Glujovsky D, et at. Epidemiology of malaria in Latin America and the Caribbean from 1990 to 2009: systematic review and meta-analysis. Value Health Reg Issues. 2015;8(3):69–79. 10.1016/j.vhri.2015.05.002 29698174

[38] 38. Cardona J. Social determinants of intestinal parasitism, malnutrition, and anemia: systematic review. Rev Panam Salud Publica. 2018;41:e143. 10.26633/RPSP.2017.143 PMC6645169 29466524

[39] 39. Cardona J. Determinantes y determinación social de la salud como confluencia de la salud pública, la epidemiología y la clínica. Arch Med. 2016;16(1):183–91.

[40] 40. Díez Roux A. La necesidad de un enfoque multinivel en epidemiología. Reg Soc. 2008;20(2):77–91.

[41] 41. Rodríguez A, López M, Harter R, Vilca L, Cárdenas R. Aspectos sociales de la malaria importada en Latinoamérica. Rev Peru Med Exp Salud Publica. 2008;25(2):208–16.

[42] 42. Wiwanitkit V. High prevalence of malaria in Myanmar migrant workers in a rural district near the Thailand–Myanmar border. Scand J Infect Dis. 2002;34:236–7. 10.1080/00365540110077272 12030408

[43] 43. Kitvatanachai S, Janyapoon K, Rhongbutsri P, Thap LC. A survey on malaria in mobile Cambodians in Aranyaprathet, Sa Kaeo Province, Thailand. Southeast Asian J Trop Med Public Health. 2003;34:48–53. 12971514

[44] 44. Chaveepojnkamjorn W, Pichainarong N. Malaria infection among the migrant population along the Thai-Myanmar border area. Southeast Asian J Trop Med Public Health. 2004;35:48–52. 15272744

[45] 45. Rodríguez C, Rivera M. Características de vivienda como factores de riesgo para malaria en un área endémica del Perú. Rev Salud UIS. 2008;40(3):197–204.

[46] 46. Barceló C. Vivienda saludable: un espacio de salud pública. Rev Cubana Hig Epidemiol. 2012;50(2):133–5.

[47] 47. Escobar F. Aspectos conceptuales para comprender la relación entre clase social y salud. En: República de Colombia, Observatorio Nacional de Salud. Clase social y salud. Bogotá: Observatorio Nacional de Salud; 2016.

[48] 48. Solar O, Irwin A. A conceptual framework for action on the social determinants of health. Social Determinants of Health Discussion Paper 2 (Policy and Practice). Geneva: World Health Organization; 2010.

[49] 49. Portes A, Hoffman K. Las estructuras de clase en América Latina: composición y cambios durante la época neoliberal. Santiago: Naciones Unidas. 2003.

[50] 50. Muntaner C, Rocha KB, Borrell C, Vallebuona C, Ibañez C, Benach J, et al. Clase social y salud en América Latina. Rev Panam Salud Publica. 2012;31(2):166–75. 22522881

[51] 51. Organización Panamericana de la Salud, Organización Mundial de la Salud. Informe de la situación de la malaria en las Américas 2014. Washington, DC: OPS; 2017.

[52] 52. Morales C, Concha S, Eslava J. Las capacidades de investigación en determinantes sociales de la salud de grupos registrado en Colciencias, Colombia (2005-2012). Rev Fac Nac Salud Publica. 2013;31(Supl 1):S126–38.

